# Low number of intrafollicular T cells may predict favourable response to rituximab-based immuno-chemotherapy in advanced follicular lymphoma: a secondary analysis of a randomized clinical trial

**DOI:** 10.1007/s00432-019-02961-9

**Published:** 2019-07-04

**Authors:** Laura Budau, Christian Wilhelm, Roland Moll, Jörg Jäkel, Carsten Hirt, Gottfried Dölken, Georg Maschmeyer, Ellen Neubauer, Konstantin Strauch, Andreas Burchert, Michael Herold, Andreas Neubauer

**Affiliations:** 1 Klinik für Hämatologie Onkologie, Immunologie, Philipps Universität Marburg, und Universitätsklinikum Gießen und Marburg, Standort Marburg, Baldingerstraße 1, 35033 Marburg, Germany; 2 Institut für Pathologie, Philipps Universität Marburg, und Universitätsklinikum Gießen und Marburg, Standort Marburg, Baldingerstraße 1, 35033 Marburg, Germany; 3 Institut für Pathologie, Universitätsklinik der RWTH Aachen, Pauwelsstraße 30, 52074 Aachen, Germany; 4 Klinik und Poliklinik für Innere Medizin Hämatologie und Onkologie, Universitätsmedizin Greifswald, Sauerbruchstraße, 17475 Greifswald, Germany; 5 Klinik für Hämatologie, Onkologie und Palliativmedizin, Klinikum Ernst von Bergmann gemeinnützige GmbH, Charlottenstraße 72, 14467 Potsdam, Germany; 6 Klinik für Gynäkologie, Philipps Universität Marburg, und Universitätsklinikum Gießen und Marburg, Standort Marburg, Baldingerstraße 1, 35033 Marburg, Germany; 7 Institut für Genetische Epidemiologie, Helmholtz Zentrum München, Ingolstädter Landstraße 1, 85764 Neuherberg, Germany; 8 Institut für medizinische Informationsverarbeitung, Biometrie und Epidemiologie Faculty of Medicine, LMU Munich, Marchioninistraße 15, 81377 München, Germany; 9 Onkologisches Zentrum, Helios-Klinikum Erfurt, Nordhäuserstr. 74, 99089 Erfurt, Germany; 10 Present Address: Kath. Marienkrankenhaus, gynäkologie Alfredstraße 9, 22087 Hamburg, Germany

**Keywords:** Follicular lymphoma, Microenvironment, Rituximab, T cells, NK cells, Macrophages

## Abstract

**Background:**

First-line rituximab therapy together with chemotherapy is the standard care for patients with advanced follicular B-cell lymphoma, as rituximab together with chemotherapy prolongs progression-free and overall survival (Herold et al. 2007; Marcus et al. 2005). However, as not all patient subgroups benefit from combined immuno-chemotherapy, we asked whether the microenvironment may predict benefit from rituximab-based therapy.

**Design:**

To address this question, we performed a retrospective immunohistochemical analysis on pathological specimens of 18 patients recruited into a randomized clinical trial, where patients with advanced follicular lymphoma were randomized into either chemotherapy or immuno-chemotherapy with rituximab (Herold et al. 2007).

**Results:**

We show here that rituximab exerts beneficial effects, especially in the subgroup of follicular lymphoma patients with low intrafollicular CD3, CD5, CD8, and ZAP70 and high CD56 and CD68 expression.

**Conclusion:**

Rituximab may overcome immune-dormancy in follicular lymphoma in cases with lower intrafollicular T-cell numbers and higher CD56 and CD68 cell counts. As this was a retrospective analysis on a small subgroup of patients, these data need to be corroborated in larger clinical trials.

**Electronic supplementary material:**

The online version of this article (10.1007/s00432-019-02961-9) contains supplementary material, which is available to authorized users.

## Introduction

Follicular lymphoma (FL) is the most frequent indolent lymphoma diagnosed. In limited stages, radiation-based therapy is thought to have curative potential. In advanced stages, when patients lack symptoms, watch and wait strategy is applied, whereas chemotherapy is the therapy of choice when treatment is needed (Yuda et al. 2016). Over decades, no progress had been seen in the therapy of advanced FL. The introduction of rituximab has changed the therapy of follicular lymphomas (FL) significantly, in that the combined immuno-chemotherapy not only prolongs progression- and event-free survival, but more importantly, overall survival (Herold et al. 2007; Marcus et al. 2008). Transformation into high-grade diffuse large B-cell lymphoma is observed in approx. 4% after 5 years in rituximab-treated patients, which seems lower than in the pre-rituximab era (Janikova et al. 2018).

Predictive factors that discriminate patients who might have a higher benefit from anti-CD20 therapy are not known. Serum concentration of APRIL or low recovery of IgG has been associated with poor survival (Kusano et al. 2018; Li et al. 2015). Using gene expression analysis, it has been shown that in FL, gene expression may separate a favourable and worse expression signature, where the favourable was characterized by a T cell, and the worse by a macrophage signature (Dave et al. 2004). As the samples were taken from FL patients not treated with rituximab, the value of these signatures in the anti-CD20 therapy era was not investigated. Different studies focused on the microenvironment in follicular lymphoma patients treated with rituximab (Carbone et al. 2009; Harjunpaa et al. 2006). One study (Harjunpaa et al. 2006) investigated R-CHOP treated patients and found different genes associated with worse progression-free survival (EPHA1, a tyrosine kinase involved in transepithelial migration, SMAD1, a transcription factor and a mediator of bone morphogenetic protein and transforming growth factor-b signalling, and MARCO, a scavenger receptor on macrophages). A recent nano-string-based analysis was able to dissect different prognostic gene sets in FL patients (Huet et al. 2018). However, the differential effect that may be caused by rituximab was not addressed in these studies.

To learn whether immune staining might play a role to predict response to rituximab-based immuno-chemotherapy, we took advantage of a randomized clinical trial where a standard chemotherapy was compared to immuno-chemotherapy (Herold et al. 2007, 2015). Thus, we compared, in a retrospective manner, the predictive role of certain immune markers in the lymph node in a small number of patients.

## Patients, materials and methods

### Patients

Patients’ data and tissue samples were obtained from four hospitals that had participated in the trial (Herold et al. 2007).^1^ In this clinical trial, patients with stage III or IV FL (grade 1 and 2) that needed treatment had been randomly assigned to either chemotherapy or chemotherapy together with rituximab. The chemotherapy consisted of mitoxantrone 8 mg/m2 intravenously (IV) on days 1 and 2; chlorambucil 3 × 3 mg/m2 orally on days 1–5; and prednisolone 25 mg/m2 orally on days 1–5 (MCP). Patients treated with R-MCP received rituximab 375 mg/m2 IV on day 1 of each therapy cycle, followed by mitoxantrone (8 mg/m2 IV) on days 3 and 4, chlorambucil (3 × 3 mg/m2 PO) on days 3–7, and prednisolone (25 mg/m2 PO) on days 3–7 (R-MCP). Patients were treated up to eight cycles (Herold et al. 2007). Lymph node material from 18 patients with follicular lymphoma stage III and IV was available for further analysis.

### Immunohistology

The immune panel is shown in Suppl. Table 2. As the expression of surface antigens in the different histological parts of FL varied distinctively, we chose to analyze the immune-profile separately for the neoplastic follicles and the extrafollicular area of the nodal lymphoma tissue, respectively. Furthermore, the overall quantity of infiltrating reactive cells in the nodal lymphoma tissue had previously proved to be an insufficient predictor of survival resulting in more specific analysis of the cell subtype and the location of infiltration in several studies (Glas et al. 2007; Wahlin et al. 2010). Immunohistochemistry was done centrally at the University Hospital Marburg, Institute of Pathology. Examination was done with a Leica DM/RB-Microscope connected to a 3CCD-camera Sony (DXC-325P) to obtain digital pictures. Intra- and extrafollicular areas were identified and from each sample two pictures were obtained from each zone. The pictures were revised and analyzed using the computerized image analysis system Image-Pro PLUS Media Cybernetics (USA), Version 4.1.0.0, and the number of cells in the field of view was calculated. The strong staining with most of the nonnuclear antibodies made individual cells difficult to discern by the Image-Pro PLUS-program, so we chose to additionally do a manual counting to get more exact results. For antigens with low/intermediate overall expression (CD3), the manual and the automatic count differed so little that every patient was sorted into the same group using the median as cut-off using the manual or automatic count, respectively. On the other hand, for antigens where the automatic count did not show representative results, i.e., antigens with very high overall expression, the manual count was used. The mean number of cells from the two pictures was used for further evaluation.

### Statistical analysis

For our analysis, we chose to analyze progression-free survival (PFS). The numbers for each antigen were separated by the median that defined the low- and the high-expression group. This was performed for the intra- as well as the extrafollicular region of the respective nodal lymphoma tissue. We compared PFS of patients with high and low expression levels for each antigen using the Kaplan–Meier method and the log-rank test. In case of lack of expression in some patients, we divided into subgroups of positive compared to missing expression. We used a criterion for nominal statistical significance of p < 0.05. The analyses were conducted using the programs “GraphPadPrism 5 for Windows” by GraphPad Software Inc, and the JMP 12 statistical software package by SAS Institute Inc., respectively. Numbers of patients at risk in the progression-free survival analyses were calculated using the JMP 12 software.

## Results

### Demographic data

The median age of these 18 patients was 59.5 years, the FLIPI score ranged from 1 to 4 at time of diagnosis. The 18 patients were evenly subdivided into the two treatment groups, one group receiving therapy with mitoxantrone, chlorambucil and prednisone (MCP) (*N* = 9), the other receiving MCP + rituximab (R-MCP) (*N* = 9). Following therapy, ten patients obtained complete remission (CR), seven went into partial remission (PR) and in one case progression during therapy was noted. Detailed patient characteristics can be found in Suppl. Table 1.

**Fig. 1:**
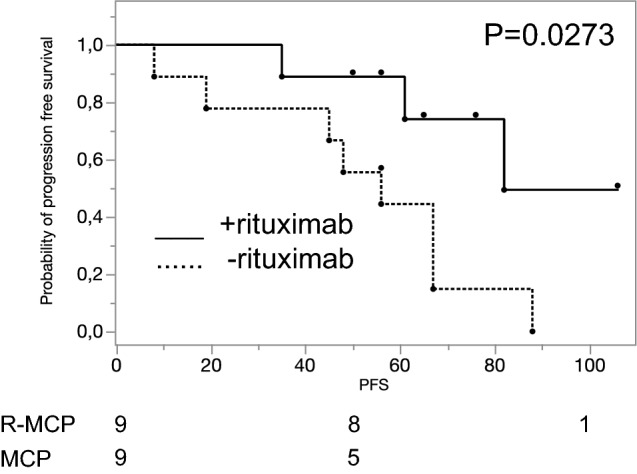
Progression-free survival (in months) of the 18 patients taken from the Herold trial (Herold et al. 2007) for further immunohistological analysis. As in the original trial employing 201 patients, the addition of rituximab induced a favourable PFS (median PFS of the nine patients treated with R-MCP as compared to the nine with MCP alone 82 vs 56 mo; *p* = 0.0273). Solid line: R-MCP; dotted: MCP. PFS is shown in months. Censored events are indicated by a dot above the line. Number of patients at risk are shown below the Kaplan–Meier plot

The median progression-free survival (PFS) for the whole group of *N* = 18 patients was 58 mo (range 8-106 mo). The median follow-up at the time of this subgroup analysis was 66 mo. At the time of this analysis, 11 patients had progressed, whereas in 7 patients remission was ongoing. First, we checked whether the small cohorts of each of the nine patients were representative for the whole study (Herold et al. 2007). Addition of rituximab resulted in a significantly prolonged PFS in the nine patients treated with rituximab as compared to the nine with MCP alone (82 mo vs 56 mo; *p* = 0.0273; Fig. 1), thus reflecting the results of the whole trial (Herold et al. 2007).

### Lower number of intrafollicular T cells may predict favourable response to rituximab

To address whether the microenvironment may be associated with a different prognosis in patients treated with rituximab, we performed immunohistochemistry using a subset of different immune markers (see suppl. Table 2). To this end, we analyzed first whether the number of T cells or CD20-positive B cells correlated with PFS in the whole group of 18 patients. This was not the case (CD3 (*p* = 0.6634), CD8 (*p* = 0.0839), ZAP70 (*p* = 0.9822)). When only considering the nine patients treated without rituximab, the number of CD3-, CD5- and ZAP70-positive T cells was—in this small cohort—not predictive of a better PFS; the only exception was intrafollicular CD8 expression; here, a higher number of CD8-positive cells was associated with favourable PFS in the nine patients not treated with rituximab (*p* = 0.045; data not shown).

**Fig. 2:**
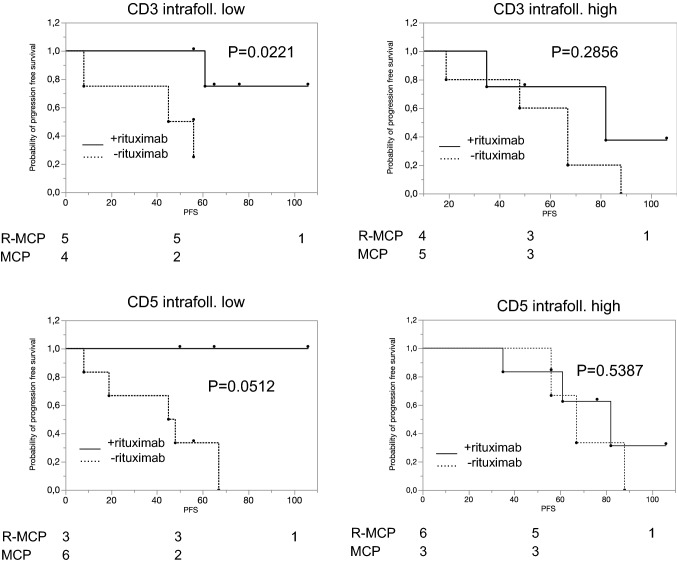

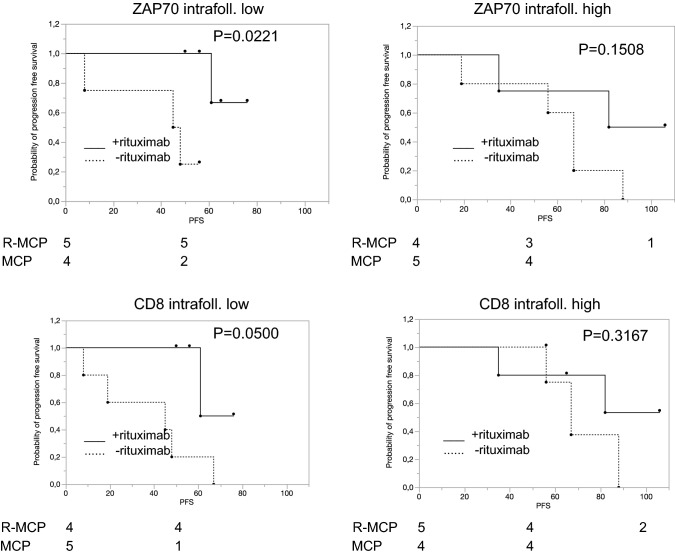
In the subgroup with a low number of intrafollicular T cells, rituximab treatment is associated with a favourable progression-free survival compared to patients treated with MCP alone. Left panel: patients whose lymphoma expressed certain T-cell markers below the median, and right panel, patients whose lymphoma expressed T-cell markers above the median. Solid line: R-MCP, dotted MCP. PFS is shown in months. Number of patients at risk are shown below the Kaplan–Meier plot

We next focused on the treatment effect of rituximab in various subgroups defined by high or low expression of a given antigen. We first asked whether the number of T cells in the neoplastic follicles correlated with a different response to rituximab. As Fig. 2 shows, this was the case for CD3, CD5, CD8 and ZAP70. When analysing intrafollicular cells, R-MCP treatment led to a more favourable PFS than MCP alone in the groups of patients with a lower density of T cell-specific antigen expression (CD3 (*p* = 0.0221), CD5 (*p* = 0.0512), CD8 (*p* = 0.0500) and ZAP70 (*p* = 0.0221)) (Fig. 2, left panel). In contrast, in patients with more intrafollicular T cells, a difference in PFS was not detected between the two groups ((CD3 (*p* = 0.2856), CD5 (*p* = 0.5387), CD8 (*p* = 0.3167), ZAP70 (*p* = 0.1508)) (Fig. 2, right panel). Representative immunohistochemical pictures illustrating CD3-positive T cells (intrafollicular vs. extrafollicular) are shown in suppl. Figure 1. The expression data of all patients, for both intra- and extrafollicular staining, are presented in Suppl. Table 3.

### Level of CD20 expression does not correlate with PFS

In both intra- and extrafollicular cells, the treatment effect of rituximab on PFS was not different for high vs. low number of CD20-positive B cells. Thus, in contrast to T-cell markers, the expression level of the common B-cell marker CD20 did not predict, in our small cohort of patients, a better response to rituximab (data not shown).

### Preferential treatment effect of rituximab with a higher number of intrafollicular CD56-positive NK cells or CD68-positive macrophages

**Fig. 3:**
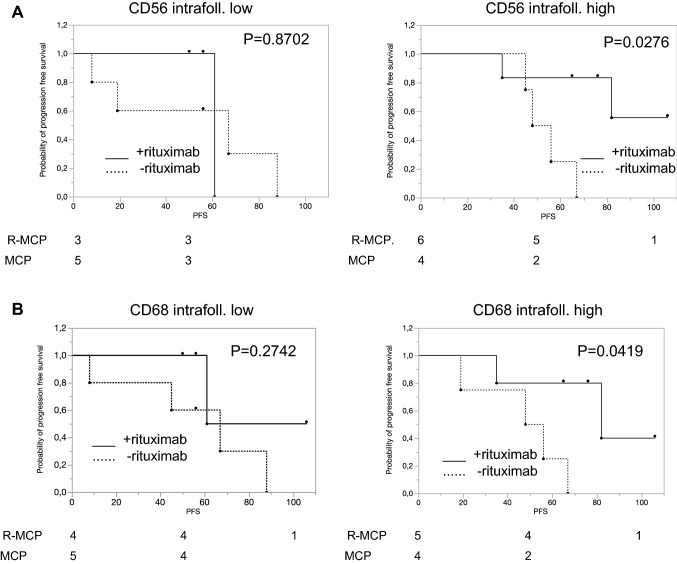
Benefit of rituximab in samples with higher number of NK cells (**a**) and macrophages (**b**). Left panel: patients with cell number below the median; right panel: patients with cell number above the median. Solid line: R-MCP, dotted MCP. PFS is shown in months. Number of patients at risk are shown below the Kaplan–Meier plot

Rituximab may activate NK cells (Weiner 2010). The subgroup with higher numbers of CD56 positive cells had a better PFS when treated with R-MCP compared to MCP alone. The median PFS in the MCP-arm was 52 months, and in the R-MCP group the median was not reached (*p* = 0.0276; Fig. 3a). This could possibly be explained by an increased antibody-dependent cellular cytotoxicity. Lastly, we analyzed expression of the macrophage marker CD68. Patients whose lymphomas revealed a high count of CD68-positive macrophages showed prolonged PFS under R-MCP; the median time to progression in the MCP-arm was 52 months vs. 82 months in the R-MCP group (*p* = 0.0419; Fig. 3b).

## Discussion

We describe here that expression of certain immune markers within the lymph nodes is associated with prolonged PFS in the R-MCP cohort, but not the chemotherapy-only group. Patients whose lymphoma tissue harbored less T-cell infiltration benefitted from the addition of rituximab compared to those who received chemotherapy only (Fig. 2). A positive effect of the addition of rituximab was not detected in the group of patients revealing T-cell numbers above the median. These data might be explained by a rituximab-induced activation of T cells, as described before (Hilchey et al. 2009; Laurent et al. 2011). Interestingly, a similar finding has been reported for the ERBB2-specific monoclonal antibody trastuzumab, used in ERBB2-positive breast cancer patients. Here, at least two studies have shown that the positive effect trastuzumab exerts with respect to PFS (and OS) was seen preferentially in the group of women that had low lymphocyte counts in their breast cancer tissue, normally a finding associated with poor prognosis (Liu et al. 2017; Perez et al. 2016). Thus, it is likely that monoclonal antibodies can convert poor immune signatures into more favourable states. Recently, a large patient cohort treated with rituximab-based therapy was analyzed with regard to the role of tumor-infiltrating lymphocytes (Xerri et al. 2017). These authors found that the higher the number of CD3 positive T cells, the better the prognosis. This is not contradictory to our findings as we did not address the role of absolute number of T cells as a prognostic marker. Rather, we asked whether patients with high vs low number of intrafollicular and extrafollicular, e.g., CD3-positive T cells benefitted differentially in the rituximab-arm vs the control arm without rituximab. According to our data, therefore, it may be likely that rituximab reverts the negative effects of lower T cell counts within the follicle in follicular lymphoma. In our small cohort, this beneficial effect was confined to the T cell counts within the follicle only.

We also analyzed the number of NK cells as well as macrophages in this study. A high amount of intrafollicular NK cells (identified by CD56) was associated with longer PFS in the rituximab cohort (*p* = 0.0276). This may point to an increased antibody-dependent cellular cytotoxicity as described previously (Fischer et al. 2006). It has also been reported that rituximab was able to activate previously hyposensitive NK cells to broaden the therapeutic effect (Du et al. 2014). In our small cohort, patients with high counts of intrafollicular macrophages showed prolonged PFS under therapy with rituximab compared to MCP alone (*p* = 0.0419), also in keeping with others (Canioni et al. 2008; Taskinen et al. 2007). In contrast, Blaker et al. found that a higher number of intrafollicular CD68-positive macrophages was associated with a higher likelihood of transformation of follicular lymphoma patients into aggressive B-cell lymphoma (Blaker et al. 2016). In their study, all patients had been treated with rituximab-based protocols. However, our study addressed the difference between a small cohort of follicular lymphoma patients treated with rituximab-based chemotherapy and compared this with the cohort treated with chemotherapy only. Both studies thus focused on different issues.

Our study suffers from small numbers, and we used a criterion of p < 0.05 for nominal statistical significance. Therefore, the data presented in this paper need to be interpreted with caution. However, the patients were balanced between the two arms of the original protocol, and the clinical outcome of the 18 patients analyzed here reflected the original trial. As the numbers are small, the data need to be confirmed in larger trials. For such future trials, we propose that the levels as determined for each antigen in the present study (see suppl. Table 3) may be applied as cut-off values since we here demonstrated that the cohort of 18 patients analyzed was representative for the whole trial (see Fig. 1). Naturally, as the chemotherapy given in this trial is not used any more (MCP), we cannot rule out that the herein observed rituximab-based immune activating effect would also be observed when using other chemotherapy regimens such as CHOP or bendamustine. It would also be interesting to apply—in addition to the markers analyzed here—the mutational load of the respective samples, as given in the m7-FLIPI (Pastore et al. 2015).

Taken together, we show here that rituximab exerts beneficial effects especially in the subgroup of follicular lymphoma patients with low intrafollicular CD3, CD5, ZAP70 and CD8, and high CD56 and CD68 expression. Although the study by Dave et al. did not use immunohistology (Dave et al. 2004), they showed that lower T cell and higher macrophage cell signature were both associated with worse prognosis in advanced follicular lymphoma. Our study, therefore, suggests that rituximab therapy could overcome the worse prognostic feature of lower T-cell and higher macrophage infiltration in follicular lymphoma. As this was a retrospective analysis on a small subgroup of patients, these data need to be corroborated in larger clinical trials. Whether rituximab resistance may be overcome by higher dosages of CD20-specific antibodies must be addressed in prospective trials. A hint could be the so-called GADOLIN trial, in which rituximab-resistant patients with indolent lymphomas were randomly assigned to either bendamustine alone or bendamustine plus obinutuzumab that was given at a significant increased dosage as compared to the previously applied rituximab (Sehn et al. 2016).

## Electronic supplementary material

Below is the link to the electronic supplementary material.

Supplementary material 1 (DOCX 7904 kb)

## Footnotes

1 The study had been registered as East German Study Group Hematology and Oncology Trial 39 and at ClinicalTrials.gov (http://www.clinicaltrials.gov/) under ID 00269113.
