# Supplementary material for: Low number of intrafollicular T cells may predict favourable response to rituximab-based immuno-chemotherapy in advanced follicular lymphoma: a secondary analysis of a randomized clinical trial
Source: J Cancer Res Clin Oncol. 2019 Jul 4;145(8):2149–56. doi: 10.1007/s00432-019-02961-9 (PMC6658576; doi:10.1007/s00432-019-02961-9)
Supplement: Supplementary file 1 — Supplementary material 1 (DOCX 7904 kb) [file 432_2019_2961_MOESM1_ESM.docx]

Suppl. Figure 1: Representative immunohistochemical analysis of follicular lymphoma cells stained with a CD3 specific monoclonal antibody, for both intrafollicular staining (A) and extrafollicular staining (B). In this particular patient (Pat-ID 8, as in suppl. Table 1 and 3), numbers were as follows: (A) 172 stained T-cells / 0.06 mm^2^ (low expression, median expression 275 T-cells / 0.06 mm^2^), (B) 585 stained T-cells / 0.06 mm^2^ (low expression, median expression 776 T-cells / 0.06 mm^2^).

Suppl. Table 1: Patient characteristics

| ***Therapy*** | ***Patient-ID*** | ***Stage*** | ***Age*** | ***Gender*** | ***FLIPI*** | ***Response*** | ***PFS*** | ***OS*** |
| --- | --- | --- | --- | --- | --- | --- | --- | --- |
| R-MCP | 1 | IV | 63 | m | 2 | CR | 106+ | 106+ |
|  | 2 | III | 65 | m | 3 | CR | 65+ | 65+ |
|  | 3 | III | 38 | x | 2 | CR | 76+ | 76+ |
|  | 4 | IV | 68 | x | 4 | PR | 50+ | 50+ |
|  | 5 | IV | 59 | m | x | PR | 82 | 100+ |
|  | 6 | III | 60 | x | 2 | PR | 56+ | 56+ |
|  | 7 | x | x | w | 2 | CR | 61 | 61 |
|  | 8 | IV | 42 | w | 4 | PR | 106+ | 106+ |
|  | 9 | IV | 62 | w | x | CR | 35 | 83 |
| MCP | 10 | x | x | w | 4 | CR | 56 | 82 |
|  | 11 | IV | 59 | x | 1 | PR | 45 | 50+ |
|  | 12 | x | x | w | 3 | PR | 67 | 73 |
|  | 13 | III | 61 | w | x | Progressive | 8 | 35 |
|  | 14 | III | 56 | m | 2 | CR | 19 | 24 |
|  | 15 | IV | 52 | m | 3 | CR | 56+ | 56+ |
|  | 16 | IV | 72 | x | 3 | PR | 88 | 88 |
|  | 17 | IV | 52 | x | 4 | CR | 48 | 60+ |
|  | 18 | x | x | w | 4 | CR | 67 | 67 |

CR: complete remission; PR: partial remission; PFS: progression free survival; OS: overall survival; +: censored at last patients visit; x: unknown

Suppl. Table 2

Monoclonal antibodies used in this study. The buffer indicated (except for CD68) refers to the heat-induced antigen retrieval procedure used.

| ***antibody*** | ***clone*** | ***origin*** | ***buffer*** | ***dilution*** |
| --- | --- | --- | --- | --- |
| **CD1a** | O10 | Immunotech | EDTA | 1:20 |
| **CD3** | SP7 | Lab Vision | EDTA | 1:300 |
| **CD5** | NCL-CD5-4C7 | Novocastra | EDTA | 1:50 |
| **CD8** | C8-144B | Dako | EDTA | 1:50 |
| **CD10** | NCL-CD10-270 | Novocastra | EDTA | 1:200 |
| **CD20** | L26 | Dako | TRS pH 6.1 | 1:600 |
| **CD56** | 123C3 | Dako | EDTA | 1:10 |
| **CD68** | PG-M1 | Dako | 0.1% Trypsin | 1:100 |
| **ZAP70** | 2F3.2 | Upstate | EDTA | 1:4000 |

Suppl. Table 3

Antigen quantification for different epitopes used in this study. The mean values (representing the number of stained cells / 0.06 mm^2^ )are given for each patient and marker. Also, the treatment arm is displayed.
